# Disrupted Regional Homogeneity in Drug-Naive Patients With Bipolar Disorder

**DOI:** 10.3389/fpsyt.2020.00825

**Published:** 2020-08-14

**Authors:** Xiaoxiao Shan, Yan Qiu, Pan Pan, Ziwei Teng, Sujuan Li, Hui Tang, Hui Xiang, Chujun Wu, Yuxi Tan, Jindong Chen, Wenbin Guo, Bolun Wang, Haishan Wu

**Affiliations:** ^1^National Clinical Research Center for Mental Disorders, and Department of Psychiatry, The Second Xiangya Hospital of Central South University, Changsha, China; ^2^Department of Psychiatry, The Third People’s Hospital of Foshan, Foshan, China; ^3^Department of Orthopedics, The Second Xiangya Hospital, Central South University, Changsha, China

**Keywords:** bipolar disorder, regional homogeneity, support vector regression, magnetic resonance imaging, bone mineral density

## Abstract

**Objective:**

Studies on alterations in the regional neural activity in the brain of patients with bipolar disorder (BD) have provided conflicting results because of different medications used and study designs. A low bone mineral density (BMD) is also observed in patients with BD. This study aimed to further explore regional neural activities in unmedicated patients with BD and their association with BMD.

**Methods:**

In this study, 40 patients with BD and 42 healthy controls were scanned through resting-state functional magnetic resonance imaging (fMRI). Imaging data were analyzed with regional homogeneity (ReHo) and pattern classification. Pearson’s correlation analyses were performed to explore the correlations between abnormal ReHo and BMD.

**Results:**

A significant increase in ReHo values in the left inferior frontal gyrus (IFG)/temporal pole, left cerebellum vermis I/vermis II/parahippocampal gyrus/brainstem, and right superior temporal gyrus (STG) and a decrease in ReHo in the occipital gyrus (OG; left middle OG/superior OG/bilateral cuneus) were found in the patients with BD (p < 0.05) compared with those in the healthy controls. No significant correlation was observed between the abnormal ReHo values in any of the brain regions of the patients with BMD.Support vector machine (SVM) analyses revealed that the ReHo values in the right STG for distinguishing patients from healthy controls showed an accuracy of 91.89%, a sensitivity of 75.68%, and a specificity of 83.78%. The ReHo values in the left cerebellum vermis I/vermis II/parahippocampal gyrus/brainstem indicated an accuracy of 78.38%, a sensitivity of 75.68%, and a specificity of 81.08%.

**Conclusion:**

This study further confirms the abnormal brain activities in extensive regions, and these brain regions are primarily located in the fronto–temporal–occipital circuit and the cerebellum vermis of patients with BD. The regional neural activity in the right STG and the left cerebellum vermis I/vermis II/parahippocampal gyrus/brainstem may serve as potential imaging markers to distinguish patients with BD from healthy controls.

## Introduction

Bipolar disorder (BD) is a chronic, debilitating mood disorder associated with a high suicide risk (1, 2). BD is characterized by its mood swings between periods of depression and manic (BD type I) or hypomanic (BD type II) episodes (3). Patients with BD can be misdiagnosed as having unipolar depression if they only experience depressive episodes with no history of mania. Such patients are at a risk of inadequate treatment and poor clinical outcomes (4, 5).

Many neuroimaging studies have been performed to improve the accuracy of the diagnosis of BD. Despite the mixed results, some brain regions have been identified to present abnormal activities and functional connectivity (FC) (6–10). Three studies have revealed the increased functional connections of the amygdala, medial prefrontal cortex, and ventrolateral prefrontal cortex and a decreased connectivity between the medial prefrontal and cingulate cortices (6–8). The enhanced connectivity of the ventral striatum seed area with the orbitofrontal cortex and amygdala has also been reported in reward processing reception in remitted BD type I (9, 10). The analytical techniques used in these neuroimaging studies are FC analysis and independent component analysis (ICA) that are efficient in exploring the function of brain networks but not local brain activities.

Regional homogeneity (ReHo) is a data-driven method to determine the degree of homogeneity within clusters through resting-state functional magnetic resonance imaging (fMRI) (11, 12). One advantage of ReHo is the ability to capture regional brain activities, compensating for the disadvantage of FC and ICA. Kendall’s coefﬁcient concordance (KCC) is commonly used in ReHo to measure the degree of the synchronization or similarity of a given voxel time series to that of its nearest neighbours through a voxel-wise method (12). An abnormal ReHo may reflect the temporal disruption of neural activities (13) and is associated with the pathophysiology of mental disorders (14). ReHo analysis has a high test–retest reliability (15) and has been applied to explore the regional abnormalities of functional synchronization in a range of psychiatric disorders, such as schizophrenia (16, 17), attention deﬁcit hyperactivity disorder (18), and depression (19).

Previous studies with ReHo in BD (20–22) revealed abnormal neural activities in extensive brain regions, including the limbic system and temporal, frontal, and parietal lobes. However, few consistent abnormalities have been reported in relation to brain regions, such as the superior temporal gyrus (STG) and the middle frontal gyrus (MFG) (20, 21). Yao et al. found an increased ReHo in the left orbital inferior frontal gyrus (OIFG) and MFG and a decreased ReHo in the IFG, insula, STG, and occipital cortex of patients with BD I and II (20). Other studies have shown that pediatric patients with BD present a decreased ReHo in the middle temporal gyrus, bilateral MFG, and medial frontal gyrus (21) and that manic and euthymic pediatric patients with BD have differences in ReHo signals in the insula, STG, and cerebellum crus I (23). In addition, a decreased ReHo in the left OFG and an increased ReHo in the right supplementary motor area, bilateral middle occipital gyrus (OG), and right precentral gyrus are observed in unmedicated patients with BD II (24). The severity of clinical symptoms in patients with bipolar depression may be correlated with the consistency in the activation levels of the right cerebellum fusiform gyrus, left insula, and cerebellar vermis-related areas (25). Several factors, including concomitant drug administration, diﬀerent BD states, types of BD, illness duration, age range, and small sample size, may have contributed to the inconsistency of these findings. Furthermore, whether ReHo is associated with clinical symptoms remains unknown.

Psychosis may be predicted on the basis of neuroanatomical biomarkers by applying multivariate pattern recognition techniques, such as support vector machine (SVM), allowing predictions at an individual level. SVM has also been applied to differentiate patients with schizophrenia (26), prodromal syndrome (27), and BD (28) from healthy controls.

Previous studies revealed that BD is associated with a relatively low bone mineral density (BMD), leading to an increased risk of fractures in patients with BD (29–31). A recent systematic review has shown an increased fracture risk (20%–80%) in patients with BD regardless of age, sex, comorbidities, and medication use (30). Nonetheless, the underlying mechanism related to the low BMD levels in BD remains unclear. Though chronic inflammation may play a role in bone loss through inflammation regulation in the central nervous system (32, 33), whether BMD is associated with the local neural activities of the brain of patients with BD has yet to be reported.

In the present study, we aimed to use ReHo to analyze the brain activity abnormalities in unmedicated patients with BD. We hypothesized that patients with BD would exhibit significantly abnormal ReHo values in specific brain regions, particularly in the prefrontal and temporal regions. We also proposed that ReHo could be employed as a potential imaging biomarker to differentiate patients with BD from healthy subjects.

## Materials and Methods

### Participants

All the patients with BD II aged 16–45 years were recruited from the Second Xiangya Hospital of Central South University, China, from March to November 2019. BD II was diagnosed by two experienced psychiatrists with the Structural Clinical Interview for Diagnostic and Statistical Manual of Mental Disorders, Fifth Edition (DSM-5) and Mini-Structured Clinical Interview for DSM-5 (SCID) and met the diagnostic criteria of BD II of DSM-5. The duration of BD II from onset did not exceed 5 years. All the patients were first diagnosed with BD II and had never taken any psychiatric medications. The inclusion criteria of BD II were as follows: 1) at least one hypomanie episode in the disease course (Criteria of “Hypomanic Episode” in DSM-5) and at least one major depressive episode in the disease course (Criteria of “Major Depressive Episode” in DSM-5); and 2) no history of a manic episode. Clinical symptoms were assessed using the Hamilton Depression Rating Scale-17 (HAMD-17), the Young Mania Rating Scale (YMRS), and the Hamilton Anxiety Rating Scale-17 (HAMA-17). The cognitive function was evaluated through the repeatable battery neuropsychologieal status (RBANS), including 12 subtests of cognitive functions; as such, the indices of delayed memory, immediate memory, language, visuospatial/constructional factors, and attention were obtained. The exclusion criteria were as follows: 1) other psychiatric disorders in accordance with the DSM-5; 2) any severe physical diseases, such as cardiovascular, kidney, or liver diseases; 3) any neuropsychiatric disorders; 4) any form of a traumatic brain injury; 5) history of electroconvulsive therapy; 6) drug or alcohol addiction; 7) pregnancy; and 8) contraindications for MRI scan.

Age-matched healthy controls were recruited from the local community through advertisements. The healthy subjects were screened using the Structured Clinical Interview for DSM-5-Nonpatient Version. The exclusion criteria were as follows: 1) any psychosis symptoms, neurological disease, or substance abuse, and 2) first-degree relatives having a history of psychiatric illness.

The study was approved by the ethics committee of the Second Xiangya Hospital of Central South University and performed in accordance with the Helsinki Declaration. All the participants provided written informed consent after a complete explanation. Additional written informed consent was obtained from the parents of patients aged below 18 years.

### BMD Measurement

The BMDs in the left hip and the lumbar spine (L1–L4), including Ward’s triangle (Ward’s), femoral neck (Neck), and trochanter (Troch) of the patients with BD were measured through dual-energy X-ray absorptiometry (Discovery Wi; S/N 87556, US).

A quantifiable indicator of osteoporosis and low bone mass (34) was used and categorized as normal (T-score of -1 and above), low bone mass (T-score between -1 and -2.5), or osteoporosis (T-score below −2.5). The entire BMD measurement was carried out in the Second Xiangya Hospital of Central South University.

### Image Acquisition and Processing

A 3.0 T Siemens scanner (Germany) was used for MRI scanning. The participants were asked to remain relaxed, supine, still, with eyes closed, and awake during the whole scanning. Foam pads and soft earplugs were used to reduce noise and scanner head motion. The scanner parameters were as follows: repetition time/echo time = 2000/30 ms, 33 axial slices, 64×64 matrix, 90° ﬂip angle, 22 cm ﬁeld of view, 4 mm section thickness, 0.6 mm slice gap, and 240 volumes.

Data were preprocessed by the Data Processing Assistant for Resting-State fMRI software (DPARSF). The ﬁrst 10 images were excluded from the analysis because of the instability of the initial MRI signal and for the participants to adapt to circumstances. The fMRI images were corrected for the acquisition delay between slices and head motion. All the subjects had no more than 2 mm of the translation in the x-, y-, or z-axes and 2° of rotation in each axis. The imaging data were then spatially normalized to the standard MNI EPI template in SPM8 and resampled to 3 mm×3 mm×3 mm. Finally, the imaging data were linearly detrended and temporally band-pass-filtered (0.01–0.08 Hz) to decrease the effect of low-frequency drifts and physiological high-frequency noise.

### ReHo Analysis

ReHo was analyzed using REST (http://resting-fmri.sourceforge.net). The ReHo maps of each participant were acquired by calculating the KCC of a given voxel time series with those of its nearest neighbours. The KCC among the voxels was divided to normalize the ReHo maps through the mean KCC of the entire brain. The Gaussian kernel of 4 mm full-width at half maximum was used to spatially smoothen the generated ReHo maps.

### Statistical Analysis

The demographics and clinical characteristics of the two groups were analyzed using two-sample—t-tests, independent t-tests, or a chi-square test when necessary. Differences in ReHo between patients and healthy controls were compared using voxel-wise two-sample t-tests with age and years of education as covariates. Gaussian random field theory was applied to correct multiple comparisons by using REST at p < 0.05 (voxel significance, p < 0.001; cluster significance, p < 0.05).

### Correlation Analysis

When significant differences in ReHo were identified in the brain regions between the two groups, the average ReHo values were extracted from these specific regions. The correlations between abnormal ReHo values and clinical parameters were determined through Pearson’s correlation analyses with the threshold for significance of p < 0.05. Bonferroni correction was employed to limit the type I error associated with multiple comparisons.

### Classification Analysis

SVM was applied to test the capacity of the extracted ReHo in any brain region to discriminate patients with BD from the controls by using the LIBSVM software package (http://www.csie.ntu.edu.tw/~cjlin/libsvm/) in MATLAB (R2013b, The MathWorks, United States). The “leave-one-out” approach was implemented in the study.

## Results

### Clinical and Demographic Characteristics

A total of 40 patients with BD II and 42 healthy controls were enrolled in this study. Of those, 37 patients with BD and 37 controls were included in the final analysis as the data of three patients and five controls were ruled out because of excessive head movements. No significant differences were observed in age, sex ratios, and years of education between the groups. The total BMD in the Total–lumbar, Ward’s, Neck, and Troch were 0.97 ± 0.10, 0.77 ± 0.12, 0.80 ± 0.09, and 0.66 ± 0.08, respectively (**Table 1**).

**Table 1:** Characteristics of the subjects.

	Patients (n=37)	Controls (n=37)	χ^2^/T	p-value
Sex (male/female)	12/25	17/20	1.418	0.234^a^
Age (years)	20.97 ± 3.07	20.84 ± 3.11	−0.188	0.851^b^
Years of education (years)	13.97 ± 1.99	14.65 ± 2.04	1.440	0.154^b^
BMD				
L1^c^	0.90 ± 0.12			
L2^c^	1.32 ± 2.04			
L3^c^	1.00 ± 0.11			
L4^c^	0.98 ± 0.10			
Total-lumbar^c^	0.97 ± 0.10			
Neck^d^	0.80 ± 0.09			
Troch^d^	0.66 ± 0.08			
Ward’s^d^	0.77 ± 0.12			
HAMD-17^e^	22.19 ± 6.86			
HAMA-17^e^	25.36 ± 8.16			
YMRS	8.08 ± 5.51			
Vocabulary learning^f^	28.53 ± 4.75			
Story retelling^f^	13.85 ± 4.59			
Immediate memory total score^e^	40.03 ± 12.67			
Graphic copy^f^	17.47 ± 1.97			
Line positioning^f^	15.88 ± 3.14			
Visual span total score^e^	31.50 ± 8.86			
Picture named^f^	8.79 ± 0.91			
Verbal fluency test^f^	19.74 ± 4.52			
Verbal function total score^e^	26.94 ± 8.10			
Digit span^f^	14.79 ± 1.77			
Coding test^f^	57.24 ± 10.48			
Attention total score^e^	68.03 ± 19.97			
Vocabulary memory^f^	7.44 ± 1.60			
Vocabulary recognition^f^	19.82 ± 0.58			
Story recall^f^	7.94 ± 2.47			
Figure memory^f^	14.62 ± 3.28			
Delayed memory score^e^	47.06 ± 12.88			
Stroop word^f^	97.76 ± 22.22			
Stroop Color^f^	69.35 ± 18.76			
Stroop Color-word^f^	41.91 ± 9.13			

^a^p values were obtained by a chi-square test.

^b^p values were obtained by two-sample t-tests.

HAMD-17, Hamilton Depression Scale-17; HAMA-17, Hamilton anxiety Scale-17; YMRS, Young Mania Rating Scale; BMD, bone mineral density; Neck, femoral neck; Troch, trochanter of femoral; Ward’s, Ward’s triangle; ^c^n=33; ^d^n=32; ^e^=36; ^f^=34.

Among the 37 remaining patients, the HAMD scores of one patient were missing. The HAMA scores of another patient were missing. Five patients failed to complete the BMD measurement.

### Differences in the ReHo Between Patients and Healthy Controls

**Figure 1:**
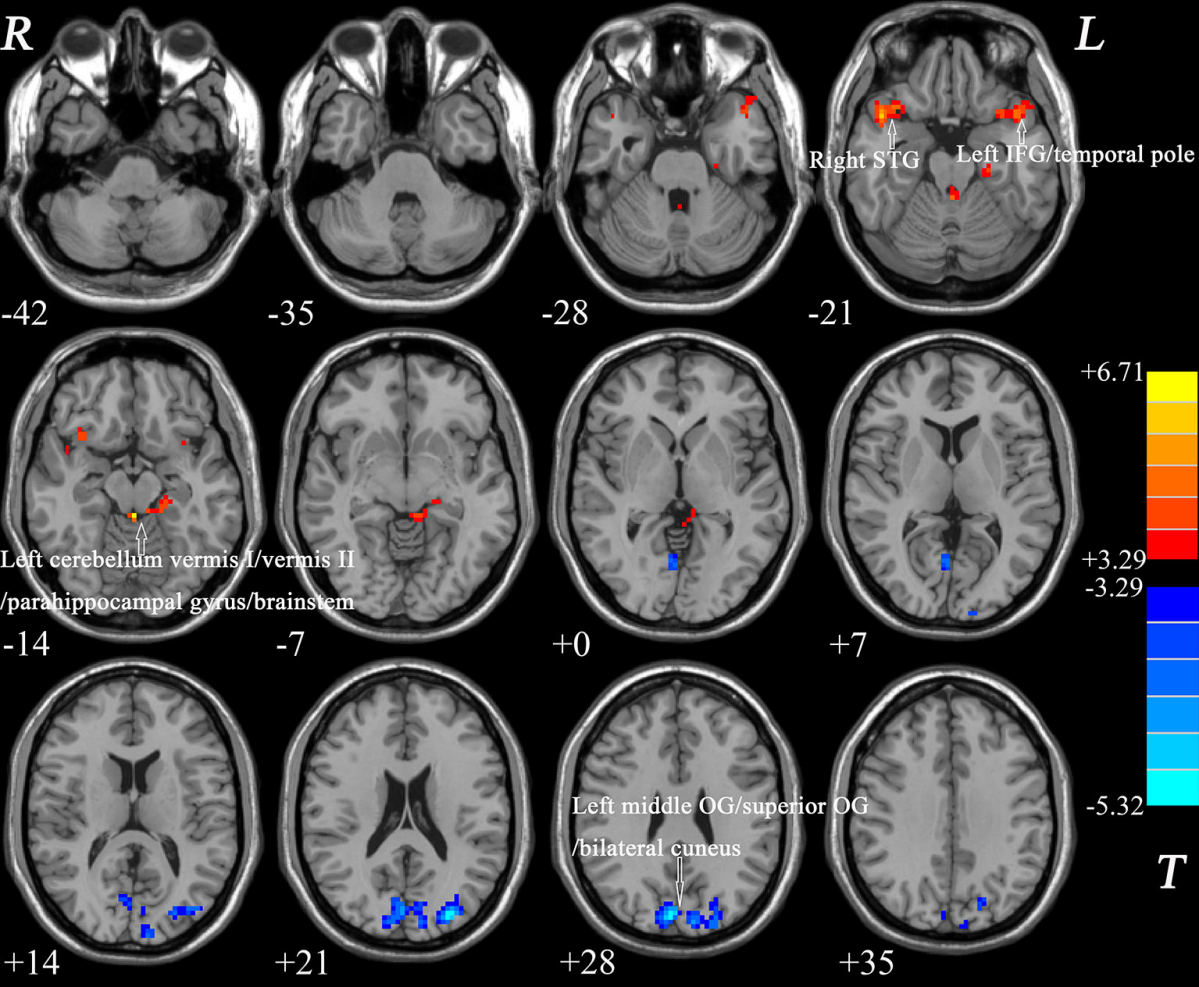
Differences in ReHo between patients and healthy controls. Increased ReHo in the left IFG/temporal pole, left cerebellum vermis I/vermis II/parahippocampal gyrus/brainstem, and right STG and a decreased ReHo in the OG (left middle OG/superior OG/bilateral cuneus) were observed in the patients with BD. The color bar represents the t values of the group analysis of ReHo. BD, bipolar disorder; ReHo, regional homogeneity; IFG, inferior frontal gyrus; STG, superior temporal gyrus; OG, occipital gyrus.

The ReHo in the left IFG/temporal pole, left cerebellum vermis I/vermis II/parahippocampal gyrus/brainstem, and right STG of the patients with BD significantly increased compared with that of the controls. By contrast, a decreased ReHo was found in the OG (left middle OG/superior OG/bilateral cuneus; **Table 2** and **Figure 1**).

**Table 2:** Alterations in ReHo among patients with BD and controls.

Cluster location	Peak (MNI)			Number of voxels	T-value
	x	y	z		
Left cerebellum vermis I/vermis II/parahippocampal gyrus/brainstem	0	−36	−15	97	6.6861
Left IFG/temporal pole	−45	15	−24	66	5.2961
Right STG	48	12	−21	61	5.6537
Left middle OG/superior OG/bilateral cuneus	−30	−84	21	393	−5.2520

BD, bipolar disorder; ReHo, regional homogeneity; IFG, inferior frontal gyrus; OG, occipital gyrus; STG, superior temporal gyrus.

The significance level was set at p < 0.05 for multiple comparisons corrected with Gaussian random field (GRF) theory (voxel significance, p < 0.001; cluster significance, p < 0.05).

### Correlation Results

**Figure 2:**
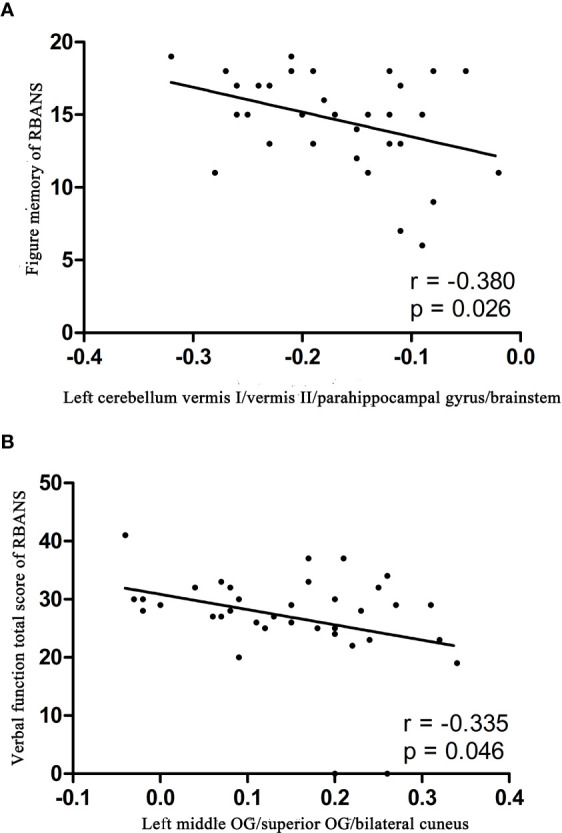
Correlations between abnormal ReHo and cognition parameter scores. **(A)** Negative correlation was observed between the ReHo values in the left cerebellum vermis I/vermis II/parahippocampal gyrus/brainstem and the figure memory of patients with BD. **(B)** Negative correlation was observed between the ReHo values in the left middle OG/superior OG/bilateral cuneus and the verbal function total score of RBANS of patients with BD. BD, bipolar disorder; ReHo, regional homogeneity; OG, occipital gyrus; RBANS, repeatable battery neuropsychological status.

The ReHo values in the left middle OG/superior OG/bilateral cuneus were negatively correlated with the verbal function total score of RBANS (r = −0.335, p = 0.046), whereas the ReHo values in the left cerebellum vermis I/vermis II/parahippocampal gyrus/brainstem were negatively correlated with the figure memory of RBANS (r = −0.380, p = 0.026) (**Figure 2**). However, these correlations were not significant after Bonferroni correction. No significant correlations were found between the ReHo values in any brain region and BMD levels of patients with BD (**Supplemental Figures**).

### SVM Analyses

**Figure 3:**
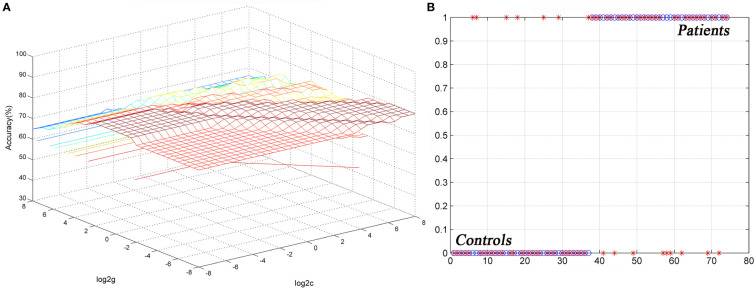
Differentiating the patients from the controls by using the increased ReHo values in the left cerebellum vermis I/vermis II/parahippocampal gyrus/brainstem. Visualization of the classifications *via* a support vector machine (SVM) by using the ReHo values in significantly different regions. **(A)** result of the SVM parameter selection *via* a 3D view; **(B)** classiﬁed map of the ReHo values in the left cerebellum vermis I/vermis II/parahippocampal gyrus/brainstem. ReHo, regional homogeneity.

**Figure 4:**
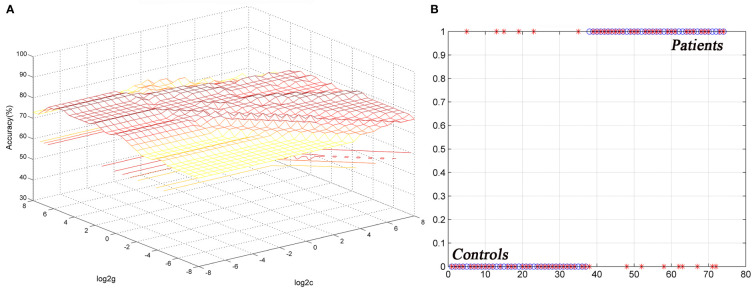
Differentiating the patients from the controls by using the increased ReHo values in the right STG. Visualization of classifications *via* a support vector machine (SVM) by using the ReHo values in the significantly different regions. **(A)** result of SVM parameter selection *via* a 3D view; **(B)** classiﬁed map of the ReHo values in the right STG. ReHo, regional homogeneity; STG, superior temporal gyrus.

The ReHo values in the left cerebellum vermis I/vermis II/parahippocampal gyrus/brainstem showed an accuracy of 78.38%, a sensitivity of 75.68%, and a specificity of 81.08% in SVM (**Figure 3**), whereas the ReHo values in the right STG showed an accuracy of 91.89%, a sensitivity of 75.68%, and a specificity of 83.78% (**Figure 4**). The detailed information of the SVM results are presented in **Table 3**.

**Table 3:** Differentiating the patients from the controls by using the ReHo values of a single region with the SVM method.

Brain region	Sensitivity	Specificity	Accuracy
Left cerebellum vermis I/vermis II/parahippocampal gyrus/brainstem	75.68% (28/37)	81.08% (30/37)	78.38% (58/74)
Right STG	75.68% (28/37)	83.78% (31/37)	91.89% (68/74)
Left IFG/temporal pole	67.57% (25/37)	72.97% (27/37)	70.27% (52/74)
Left middle OG/superior OG/bilateral cuneus	62.16% (23/37)	83.78% (31/37)	72.97% (54/74)

ReHo, regional homogeneity; SVM, support vector machines; IFG, inferior frontal gyrus; OG, occipital gyrus; STG, superior temporal gyrus.

## Discussion

This study found abnormal brain activities in patients with BD regarding ReHo and explored the relationship between the brain activity of specific regions and BMD levels. The results showed that the ReHo values in the left cerebellum vermis I/vermis II/parahippocampal gyrus/brainstem, left IFG/temporal pole, and right STG increased, and the ReHo in the OG (left middle OG/superior OG/bilateral cuneus) decreased. However, ReHo and BMD in any brain region were not significantly correlated. Furthermore, the SVM result showed that the ReHo values in the right STG and left cerebellum vermis I/vermis II/parahippocampal gyrus/brainstem regions might be potential imaging markers to distinguish patients from healthy controls.

The frontal cortex has an established effect on emotional regulation, mental flexibility, impulsivity, self-awareness, and self-monitoring (35). As part of the frontal cortex, the ventrolateral prefrontal cortex (including IFG) (7) and the orbitofrontal cortex (24) are also implicated in the pathophysiological features of BD. The IFG is considered to be crucially involved in the adjustment of emotional intensity and the integration of emotional information (36). Previous studies revealed the abnormal FC between the IFG and the medial prefrontal cortex, amygdala, and STG of patients with BD (8, 37). The abnormal patterns of the FC between the IFG and nodes of the default network may also be associated with cognitive symptoms found in patients with BD (38). The functional dysconnectivity of the IFG with emotion-regulating regions, as a characteristic abnormality of BD, can assist in clinical diagnosis (8). Our study further provided evidence on disturbed regional activities in IFG, suggesting the critical role of the ventrolateral prefrontal cortex in the neurological mechanism of BD.

The temporal gyrus, as an auditory and visual-related brain region, is involved in the processing of facial emotions and working memory (39, 40). Structural and functional neuroimaging studies have revealed that BD is also associated with functional changes in the temporal gyrus (41). A decreased gray matter volume in the temporal gyrus has been reported in adult patients with BD (41). Several functional neuroimaging studies have indicated the dysfunction in related neural networks, which include the temporal gyrus, in emotional processing deficits (42, 43). A decreased connectivity between the amygdala and the temporal gyrus during an emotional face judgment task is found in pediatric patients with BD (44). Furthermore, the decreased ReHo values in the temporal gyrus have also been reported in patients with BD (20, 21). The ReHo values obtained from the temporal gyrus were significantly different between patients with BD and unipolar depression; this result suggested that ReHo values could be a potential neuroimaging marker of BD (20). Our results implied that the right temporal gyrus was presumably a part of the correlative functional network associated with BD.

In this study, the ReHo values in the occipital lobe decreased, suggesting a decrease in the local synchronization of spontaneous neural activities in the OG. OG has been revealed to be responsible for emotional facial adjustment as the main visual processing center (45, 46), particularly the middle OG that promotes interpersonal communication in specific social environments (47). In pediatric patients with BD, the amplitude of the low-frequency fluctuation decreases in the bilateral inferior OG and left precuneus (48). The ReHo values in the OG, insula, and temporal cortex of patients with BD I or BD II also decrease, and this finding is consistent with our results (20). Structural MRI studies have revealed a decrease in the volume and thickness of the occipital cortex in patients with BD (11, 48). Moreover, they showed that the volume of the gray matter in the cuneus is associated with the performance of an inhibitory control task in patients with BD (49). Task-related fMRI results have also demonstrated the abnormal activation of OG in patients with BD when they are asked to perform tasks involving emotional images (50), emotional face encoding (51), and sensory processing (52). Hence, the decreased ReHo values in OG found in this study might be associated with the emotional deﬁcits commonly found in patients with BD.

The ReHo in the cerebellum vermis cortex, including vermis I/vermis II, increased. Previous studies also reported abnormalities in the cerebellum cortex of patients with BD (23, 25), suggesting that the cerebellum might also play a significant role in the pathophysiological features of BD (53). The cerebellum is interconnected with other prefrontal and limbic brain regions and may work cooperatively to regulate cognitive and emotional processing (54). Xiao et al (23) found an increase in ReHo in the right cerebellum crus1 in pediatric patients with BD, and this observation also agrees with our finding about abnormalities in the cerebellum vermis.

A previous study revealed that a specificity or sensitivity greater than 0.7 is conducive to the establishment of diagnostic indicators (55). In contrast, a specificity or sensitivity of less than 0.6 may indicate a poor establishment of diagnostic indicators (56). SVM has been widely used in biomedical diagnoses (57). A valid radiomic method through resting-state fMRI can identify patients with BD from healthy controls with more than 80% of classification accuracy *via* SVM analysis (28). Therefore, this method is a potential adjunctive tool for clinical diagnostic systems. The SVM analysis in our study showed that the accuracy, sensitivity, and specificity for distinguishing patients from healthy controls were more than 0.7 in terms of the ReHo values of the right STG regions and the left cerebellum vermis I/vermis II/parahippocampal gyrus/brainstem. Hence, the ReHo values in these brain regions could be employed as potential imaging markers to distinguish patients with BD from controls.

Our study also tried to explore the correlation between abnormal ReHo and BMD in BD. However, abnormal ReHo was not correlated with BMD in patients with BD. Therefore, the potential mechanism underlying the low BMD of patients with BD remains unknown. Aspects such as inﬂammatory cytokines, endocrine factors, oxidative stress, and mitochondrial dysfunction have common pathways that can influence neuroprogression and lead to a low BMD in BD (58, 59). Chronic neuroinflammation results in an increased level of free radicals, lipid peroxidation, reduced mitochondrial function, and excitatory toxicity, and all of them have neuroprogressive effects because of neurotoxicity (60). No significant correlation was found between ReHo and BMD possibly because of a relatively small sample size, and/or a low signal-to-noise ratio in resting-state fMRI.

Apart from the small sample size, other limitations in the current study should be considered. First, the abnormalities in gray/white matters were not evaluated, but whether these abnormalities were a basis of changes in ReHo remains unclear. Second, the use of the Montreal Neurological Institute template might be a potential confounding factor as the template was initially generated on the basis of a Caucasian population, so it might not fit the study on the Chinese population. Third, the resolution in our study was relatively lower than that of other resting-state fMRI studies, which might have limited the interpretation of the fMRI results. Lastly, ReHo was only limited to activity in local brain regions, failing to explore functional connections between whole brain regions. As a voxel-based method, its objectivity might cause potential registration or normalization errors. Hence, the present results should be interpreted with caution.

In conclusion, this study provided supporting evidence on disrupted regional neural activities in extensive regions and these brain regions primarily locate in the fronto–temporal–occipital circuit and the cerebellum vermis in patients with BD II during the resting state. The regional neural activity in the right STG and the left cerebellum vermis I/vermis II/parahippocampal gyrus/brainstem might serve as potential imaging markers to distinguish patients with BD from healthy controls.

Future research should continue to examine patients with BD in large sample sizes through functional imaging and longitudinal studies to clarify the potential causal relationship between changes in ReHo and BD.

## Data Availability Statement

All datasets presented in this study are included in the article/**Supplementary Material**.

## Ethics Statement

The studies involving human participants were reviewed and approved by: The study was approved by the ethics committee of the Second Xiangya Hospital of Central South University. Our study was performed in accordance with the Helsinki Declaration. All the participants provided written informed consent. Additional written informed consent was obtained from the parents of patients aged below 18 years.

## Author Contributions

All authors contributed to the article and approved the submitted version. XS and YQ contributed equally to this work, wrote the manuscript, and conducted the study. ZT, SL, HT, HX, CW, and YT collected the original imaging data. BW and HW designed the study. WG and JC managed and analyzed the imaging data.

## Funding

This study was supported by grants from the National Natural Science Foundation of China (Grant Nos. 81971258 and 81270019).

## Conflict of Interest

The authors declare that the research was conducted in the absence of any commercial or financial relationships that could be construed as a potential conflict of interest.
