# Supplementary material for: Disrupted Regional Homogeneity in Drug-Naive Patients With Bipolar Disorder
Source: Front Psychiatry. 2020 Aug 14;11:825. doi: 10.3389/fpsyt.2020.00825 (PMC7456987; doi:10.3389/fpsyt.2020.00825)

**Supplemental Figures**

**Figure S1** Correlations between increased ReHo value in left cerebellum vermis I/vermis II/parahippocampa gyrus/brainstem and BMD

**Figure S2** Correlations between increased ReHo value in left IFG/temporal pole and BMD

**Figure S3** Correlations between increased ReHo value in right STG and BMD

**Figure S4** Correlations between decreased ReHo value in left middle OG/superior OG /bilateral cuneus and BMD

**Supplemental Tables**

The extracted ReHo in abnormal brain regions was analyzed using regions of interest (ROIs). The mean time series of ROI with extracted ReHo in brain regions and the mean time series of all voxels in the brain were analyzed by Pearson correlation analysis to obtain the correlation coefficient r for every two regions of interest. Then, r is normalized by using Fisher Z-conversion to convert r into Z points, and Z images represent the functional connection strength of brain regions. Two independent sample T-tests were used to explore the difference in Z images between the patients group and the healthy control group.

**Table S1** The different brain regions of whole brain functional connection seed-based on the left cerebellum vermis I/vermis II/parahippocampa gyrus/brainstem

| Cluster location | Peak (MNI) | | | Number of voxels | *T* value^a^ |
| --- | --- | --- | --- | --- | --- |
|  | x | y | z |  |  |
| Left Brainstem/CerebellumVermis III | 3 | -36 | -12 | 21 | 6.6911 |
| Right Fusiform | 39 | -39 | -18 | 75 | -4.6982 |

**Table S2** The different brain regions of whole brain functional connection seed-based on the left IFG /temporal pole

| Cluster location | Peak (MNI) | | | Number of voxels | T value^a^ |
| --- | --- | --- | --- | --- | --- |
|  | x | y | z |  |  |
| Right Cerebellum Posterior Lobe/Cerebellum CrusⅡ | 42 | -75 | -45 | 38 | 3.5485 |
| Right Superior Frontal Gyrus | 15 | 30 | -19 | 52 | -4.5492 |
| Left Superior Frontal Gyrus | -15 | 27 | -18 | 28 | -4.8057 |

IFG=inferior frontal gyrus

**Table S3** The different brain regions of whole brain functional connection seed-based on the left middle OG/superior OG /bilateral cuneus

| Cluster location | Peak (MNI) | | | Number of voxels | T value^a^ |
| --- | --- | --- | --- | --- | --- |
|  | x | y | z |  |  |
| Right Fusiform/Parahippocampa Gyrus | 36 | -36 | -18 | 115 | -5.3335 |
| Right Precuneus | 9 | -60 | 57 | 50 | -4.5992 |

OG=occipital gyrus

**Table S4** The different brain regions of whole brain functional connection seed-based on the right STG

| Cluster location | Peak (MNI) | | | Number of voxels | T value^a^ |
| --- | --- | --- | --- | --- | --- |
|  | x | y | z |  |  |
| Right Cerebellum Posterior Lobe/Cerebellum VIII | 24 | -66 | -48 | 28 | -4.2905 |
| Right Superior Frontal Gyrus | 24 | 39 | -21 | 38 | -4.3498 |

STG=superior temporal gyrus

**Figure S1**


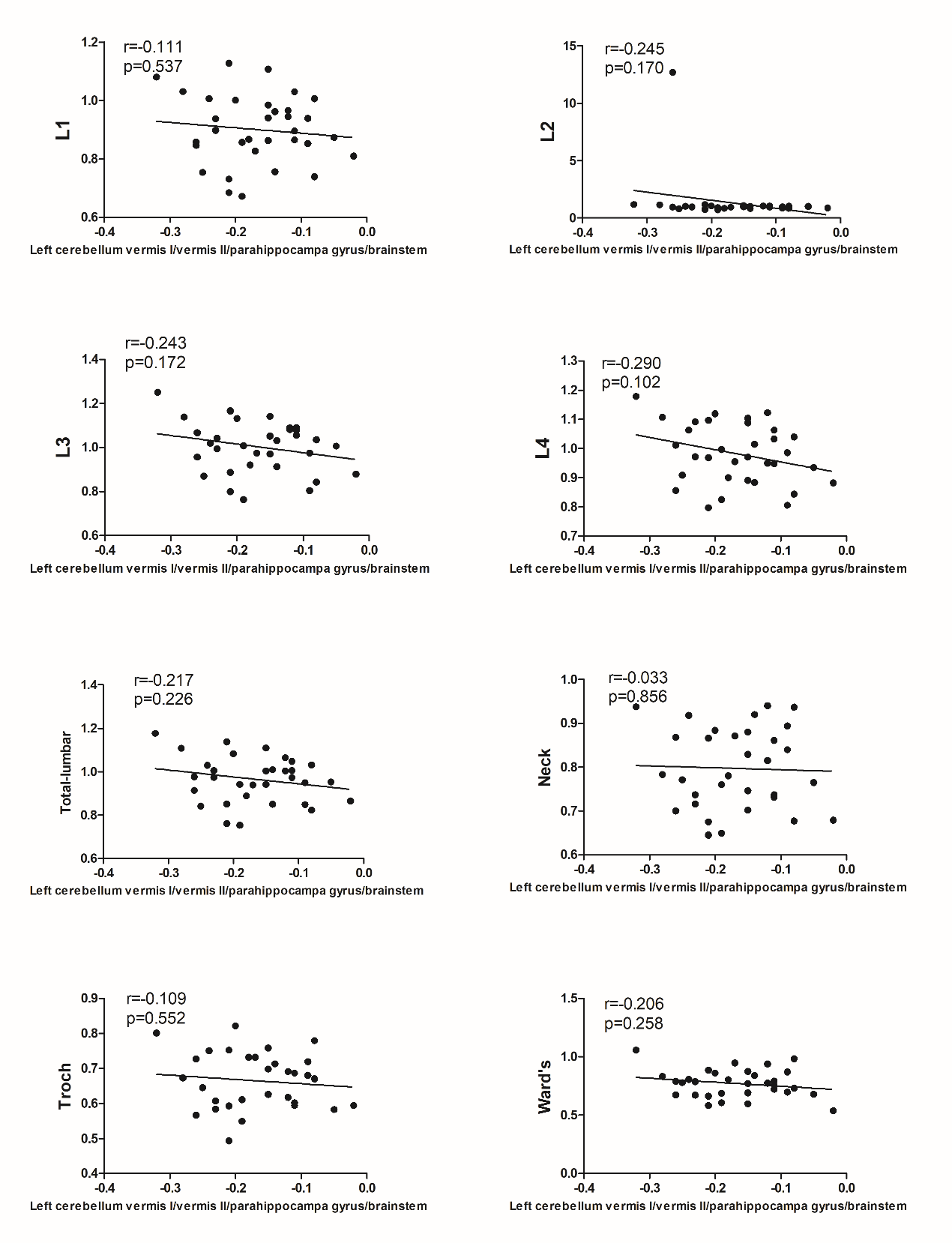


**Figure S2**


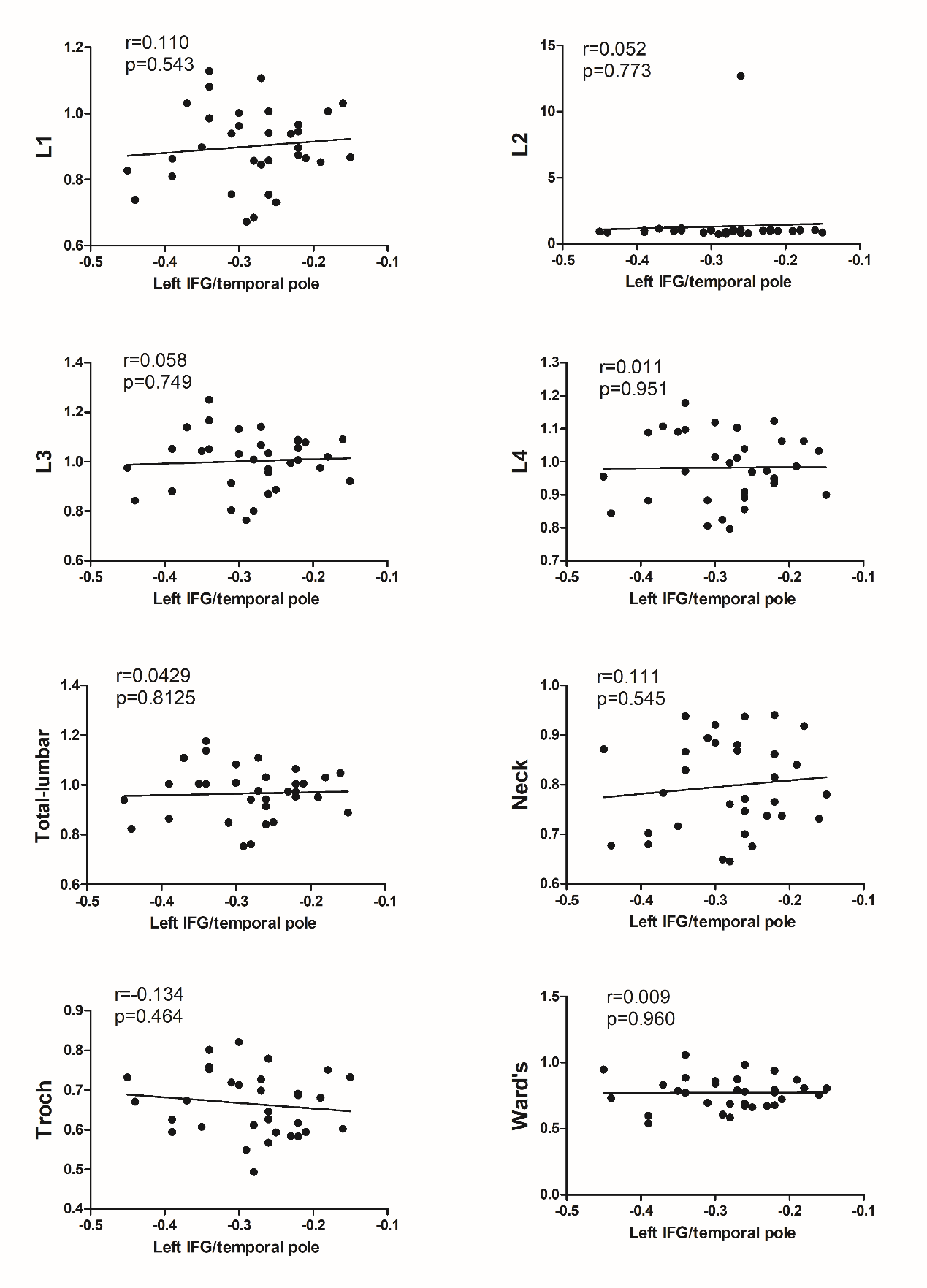


**Figure S3**


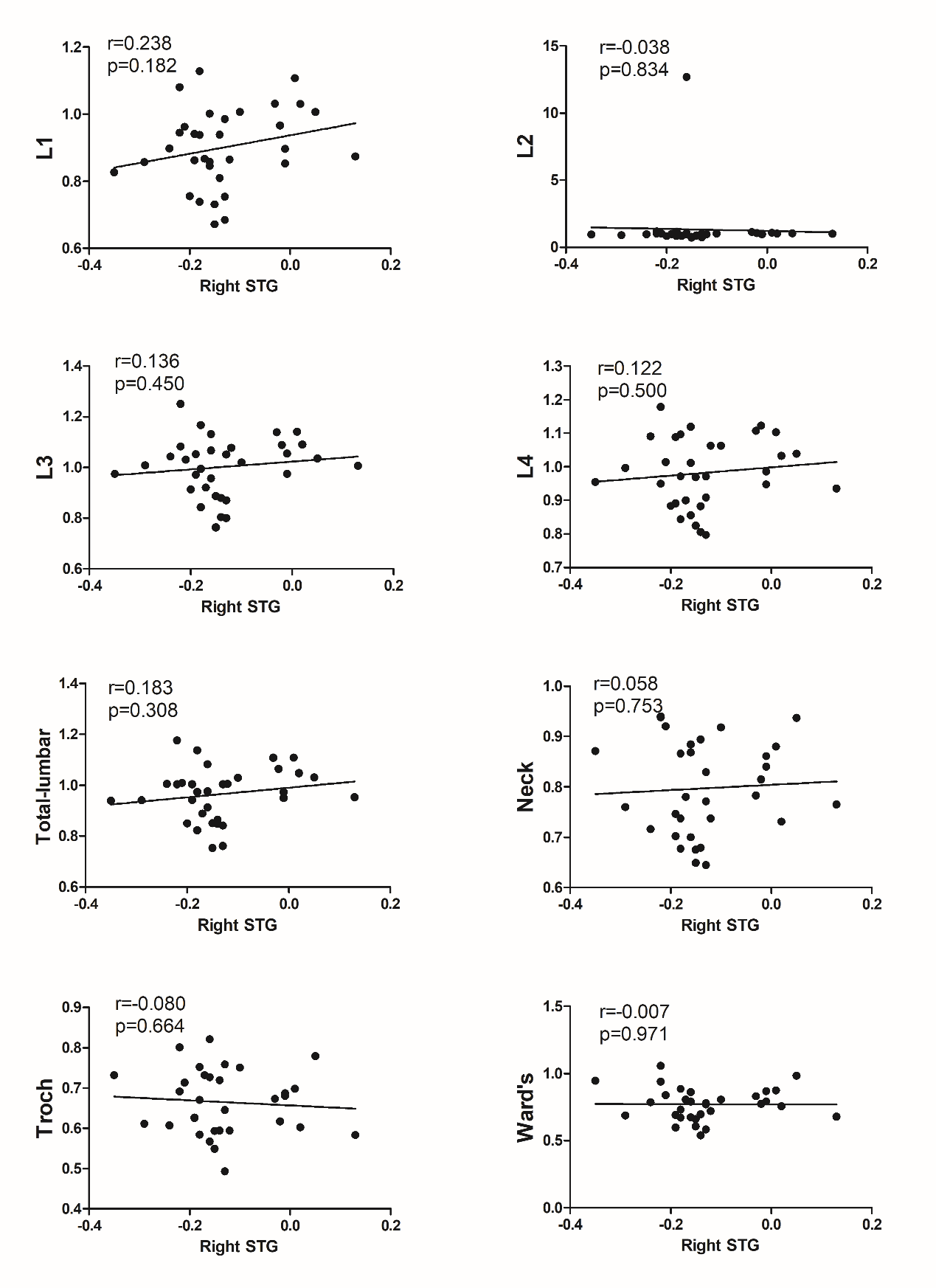


**Figure S4**


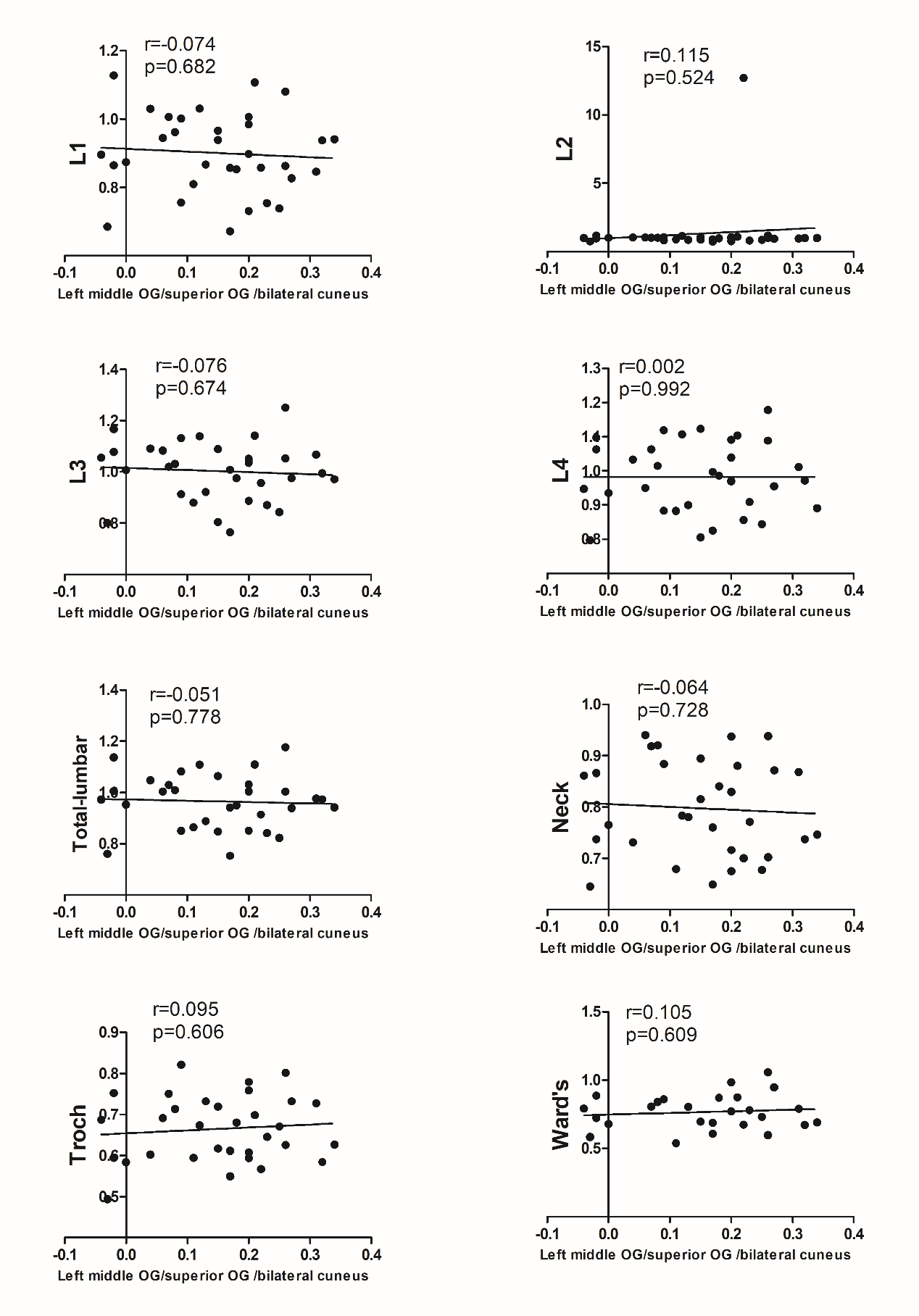

Supplement: Supplementary file 1 [file Table_1.docx]
